# Profiling of differentially expressed circRNAs and functional prediction in peripheral blood mononuclear cells from patients with rheumatoid arthritis

**DOI:** 10.1080/07853890.2022.2156596

**Published:** 2023-01-20

**Authors:** Li Xue, Biao Wang, Jianhong Zhu, Qian He, Fang Huang, Wei Wang, Li Tao, Yan Wang, Nan Xu, Ni Yang, Li Jin, Hua Zhang, Ning Gao, Ke Lei, Yanping Zhang, Chaoliang Xiong, Jing Lei, Ting Zhang, Yan Geng, Ming Li

**Affiliations:** a Department of Clinical Laboratory, The Second Affiliated Hospital of Xi’an Jiaotong University, Xi’an, China; b Clinical Research Center for Endemic Disease of Shaanxi Province, Xi’an, China; c Department of Immunology and Pathogenic Biology, Health Science Center, Xi’an Jiaotong University, Xi’an, China; d Department of Bone and Joint Surgery, The Second Affiliated Hospital of Xi’an Jiaotong University, Xi’an, China; e Emergency Department, The Second Affiliated Hospital of Xi’an Jiaotong University, Xi’an, China; f National Local Joint Engineering Research Centre of Biodiagnostics and Biotherapy, The Second Affiliated Hospital of Xi’an Jiaotong University, Xi’an, China; g Department of Cardiovascular Surgery, The First Affiliated Hospital of Xi’an Jiaotong University, Xi’an, China

**Keywords:** Rheumatoid arthritis, circular RNAs, expression profile, peripheral blood mononuclear cells, microarray

## Abstract

**Background:**

Rheumatoid arthritis (RA) is a chronic autoimmune disease associated with an increased risk of death, but its underlying mechanisms are not fully understood. Circular RNAs (circRNAs) have recently been implicated in various biological processes. The aim of this study was to investigate the key circRNAs related to RA.

**Methods:**

A microarray assay was used to identify the differentially expressed circRNAs (DEcircRNAs) in peripheral blood mononuclear cells (PBMCs) from patients with RA compared to patients with osteoarthritis (OA) and healthy controls. Then, quantitative real-time PCR was applied to verify the DEcircRNAs, and correlations between the levels of DEcircRNAs and laboratory indices were analysed. We also performed extensive bioinformatic analyses including Gene Ontology (GO), Kyoto Encyclopedia of Genes and Genome (KEGG) pathway and potential circRNA–miRNA–mRNA network analyses to predict the function of these DEcircRNAs.

**Results:**

A total of 35,342 and 6146 DEcircRNAs were detected in RA patients compared to controls and OA patients, respectively. Nine out of the DEcircRNAs in RA were validated by real-time PCR. There were correlations between the levels of DEcircRNAs and some of the laboratory indices. GO analyses revealed that these DEcircRNAs in RA were closely related to cellular protein metabolic processes, gene expression, the immune system, cell cycle, posttranslational protein modification and collagen formation. Functional annotation of host genes of these DEcircRNAs was implicated in several significantly enriched pathways, including metabolic pathways, ECM–receptor interaction, the PI3K–Akt signalling pathway, the AMPK signalling pathway, leukocyte transendothelial migration, platelet activation and the cAMP signalling pathway, which might be responsible for the pathophysiology of RA.

**Conclusions:**

The findings of this study may help to elucidate the role of circRNAs in the specific mechanism underlying RA.

**Key messages**
- Microarray assays showed that a total of 35,342 and 6146 DEcircRNAs were detected in RA patients compared to controls and OA patients, respectively.
- Nine out of the DEcircRNAs in RA were validated by real-time PCR, and the levels of the DEcircRNAs were related to some of the laboratory indices.
- GO analyses revealed that the DEcircRNAs in RA were closely related to cellular protein metabolic processes, gene expression, the immune system, etc.
- Functional annotation of host genes of the DEcircRNAs in RA was implicated in several significantly enriched pathways, including metabolic pathways, ECM–receptor interaction, the PI3K–Akt signalling pathway, etc.

## Introduction

Rheumatoid arthritis (RA) is a chronic autoimmune disease characterized by synovial hyperplasia, pannus formation and joint damage that affects approximately 0.5–1% of adults worldwide [1]. A recent study reported that despite decreasing mortality rates due to advancements in RA management in recent years, RA continues to be linked to an increased risk of death [2]. Moreover, although major progress in the diagnosis and treatment of RA has been made in recent years, damage to articular cartilage and bone and long-term disability in RA patients are still common [3]. The pathogenesis of RA is complex and involves environmental factors that trigger disease in genetically susceptible individuals [4]. The most effective therapeutic approach of RA requires early diagnosis and adoption of an optimal nonpharmacological and pharmacological treatment, associated with periodic evaluation of therapeutic efficacy and safety [5]. Therefore, it is necessary to comprehensively elucidate the inflammatory and immunoregulatory mechanisms that underlie the etiopathogenesis of RA [6].

As a new class of endogenous noncoding RNAs, circular RNAs (circRNAs) have recently attracted more attention due to their pivotal role in the modulation of gene expression at the transcriptional and posttranscriptional levels [7]. Accumulating studies have implicated the biological functions of circRNAs such as microRNA (miRNA) sponging, regulating target gene transcription and forming RNA–protein complexes [8,9]. Importantly, several studies have also demonstrated that circRNAs may be involved in the process of mediating inflammation and immune regulation in autoimmune diseases [10–12]. A number of circRNAs have been shown to be aberrantly expressed in RA patients and may be involved in the pathogenesis of RA [13–15]. Although these lines of evidence indicate that circRNAs have the potential to serve as novel diagnostic biomarkers and therapeutic targets for RA, the expression profiles and biological functions of circRNAs in RA remain elusive.

We aimed to investigate the profiles of circRNA expression in RA in the Northwest Chinese Han population. We used a circRNA microarray assay to identify the differentially expressed circRNAs (DEcircRNAs) in peripheral blood mononuclear cells (PBMCs) from patients with RA compared to healthy controls and patients with osteoarthritis (OA). Then, quantitative real-time PCR (qRT-PCR) was applied to verify the DEcircRNAs. We next performed extensive bioinformatic analyses including Gene Ontology (GO), Kyoto Encyclopedia of Genes and Genome (KEGG) pathway and potential circRNA–miRNA–mRNA network analyses, to predict the function of DEcircRNAs, and to reveal the possible pathogenesis of RA. Our results provide a novel perspective for the pathogenesis and diagnostic markers of RA.

## Methods

### Study population

Patients with RA were selected from the Department of Rheumatology and Immunology of the Second Affiliated Hospital of Xi’an Jiaotong University during the period from January 2019 to December 2020. Age- and gender-matched healthy subjects and patients with OA were from the Health Examination Center and the Department of Orthopaedics of our hospital, respectively. Both healthy subjects and OA patients were recruited as the controls of RA patients. RA patients fulfilled the criteria of the 2010 American College of Rheumatology (ACR). Patients with RA and OA were excluded if they had other autoimmune diseases, haematologic diseases, malignancies or infections; had any history of other chronic diseases, such as diabetes mellitus, dyslipidaemia, thyroid dysfunction, or severe liver or kidney impairment; or received corticosteroid treatment within the last 3 months. Fresh circulating blood samples (4 mL) were collected from all of the participants, and serum was collected and stored at –80 °C. This study was performed in accordance with the Declaration of Helsinki and was approved by the Research Committee of Human Investigation of Xi’an Jiaotong University Health Science Center (2018-1602), and written informed consent of the study was obtained from all participants.

### Peripheral blood mononuclear cell isolation and RNA extraction

Peripheral venous blood samples were collected from all participants. Peripheral blood mononuclear cells were isolated by density gradient centrifugation using Lymphocyte Separation Medium (TBD, Tianjin, China), and were stored at –80 °C in TRIzol (Invitrogen, Carlsbad, CA). Total RNA was extracted from PBMCs using TRIzol reagent and purified with a Qiagen RNA easy Mini Kit (Qiagen, Hilden, Germany) according to the manufacturer’s instructions. The concentration and purity of RNA were determined using a NanoDrop ND-1000 spectrophotometer (Thermo Fisher Scientific, Waltham, MA). The integrity of RNA was assessed by electrophoresis on a denaturing agarose gel.

### Microarray profiling and data analysis

The expression profiles of circRNA were detected by Agilent human circRNA Array V2.0 according to the recommended procedures provided by CapitalBio Technology Corporation (Beijing, China). The assay included the process of RNA labelling, hybridization, scanning and data analysis. Raw data were extracted and preprocessed by using Feature Extraction software V10.7 (Agilent Technologies, Santa Clara, CA). Then, normalization, quality control and differential analysis of the data were performed using Agilent Gene Spring V13.0 software. Fold-change ≥ 2.0 and a *p* value <0.05 were considered the criteria for identifying differentially expressed genes.

### qRT-PCR assay validation

qRT-PCR assays were performed to validate the DEcircRNAs that were detected in the microarray analysis. Total RNA from PBMCs was extracted using TRIzol reagent (Ambion, Austin, TX) according to the manufacturer’s protocol. The quantity of RNA was determined using a NanoDrop2000 (Nano Drop Products, Wilmington, DE). cDNA was synthesized using Evo M-MLV RT Premix (AG11706, ACCURATE BIO-TECHNOLOGY, Hunan, China). The qRT-PCR assay was conducted using a SYBR^®^ Green Premix Pro Taq HSqPCR Kit (AG11701, ACCURATE BIOTECHNOLOGY, Hunan, China) in an ABI Prism 7500 Real-Time PCR System (Applied Biosystems, Foster City, CA). The comparative CT (2^–ΔΔCT^) method was used to obtain the fold change in circRNA expression levels. All experiments were performed in triplicate.

### Bioinformatic analysis of the differentially expressed circRNAs

CircRNAs act as competing endogenous RNAs and play an important role in miRNA-mediated posttranscriptional gene regulation by binding with the corresponding miRNAs. Target miRNAs of the DEcircRNAs were predicted by using MiRanda (http://www.microrna.org/microrna/home.do), RNA hybrid databases (https://bibiserv.cebitec.uni-bielefeld.de/rnahybrid) and StarBase (http://starbase.sysu.edu.cn/).

The interaction between miRNAs and mRNAs was predicted based on MiRanda and TargetScan (http://www.targetscan.org). The visual circRNAs-miRNAs or circRNAs–miRNAs–mRNAs regulatory networks were established by using Cytoscape software (http://www.cytoscape.org). The function of these target genes, including biological process (BP), molecular function (MF) and cellular component (CC) were defined by GO (http://www.geneontology.org). Kyoto Encyclopedia of Genes and Genomes analysis was also performed to illuminate their involved biological pathways.

### Statistical analysis

Continuous parameters were described as the means ± standard deviations and qualitative parameters as numbers (%). The differences in continuous variables were analysed using one-way analysis of variance or Student’s *t*-test, while Chi-squared tests were performed to compare the differences in qualitative variables. The correlations between DEcircRNAs and Laboratory indices were analysed by Spearman’s correlation test. All these statistical analyses were performed using SPSS software (version 16.0, Chicago, IL) with *p* < 0.05 considered statistically significant.

## Results

### Baseline characteristics of the study subjects

This study included a total of 55 patients with RA, 55 patients with OA and 55 controls. The demographic characteristics of the subjects are given in Table 1. There were no differences with regard to age, sex or BMI among the three groups for either the sequencing cohort or the validation cohort.

**Table 1.** Demographic characteristics of the study subjects.

	Sequencing cohorts				Validation cohorts			
	Controls (*n* = 3)	RA (*n* = 3)	OA (*n* = 3)	*p* Value	Controls (*n* = 52)	RA (*n* = 52)	OA (*n* = 52)	*p* Value
Age (years ± SD)	53.16 ± 4.63	56.48 ± 6.12	57.86 ± 6.71	0.627	54.31 ± 6.15	55.42 ± 9.67	56.92 ± 7.32	0.238
Gender (F/M)	2/1	2/1	2/1	1.000	36/16	37/15	33/19	0.682
BMI (kg/m^2^)	25.1 ± 3.6	26.3 ± 3.2	25.8 ± 3.1	0.907	26.5 ± 3.8	27.1 ± 4.1	26.9 ± 3.3	0.708

F: female; M: male; BMI: body mass index.

### Overview of circRNAs expression profiles

**Figure 1.**
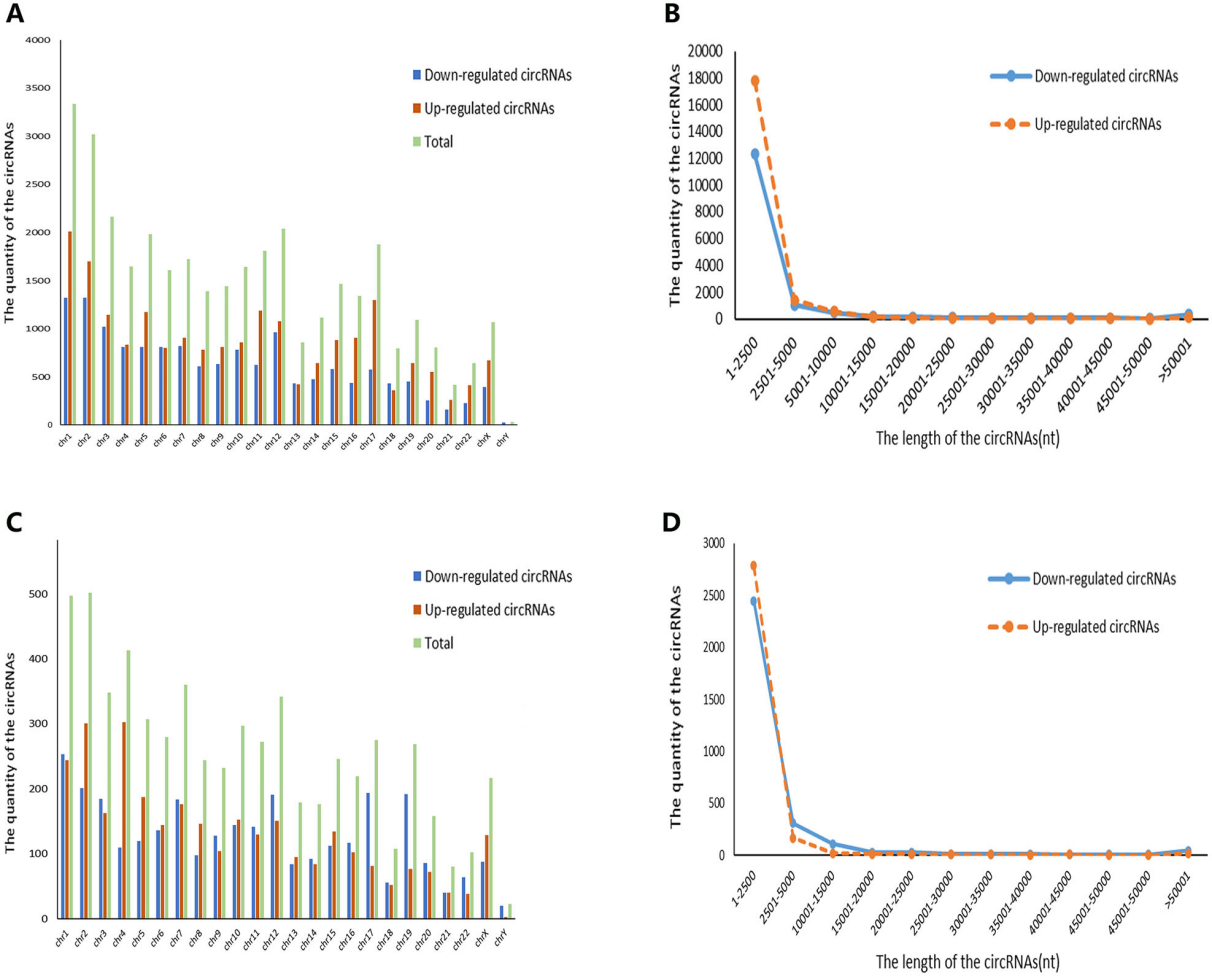
Chromosomal distribution of differentially expressed circRNAs in PBMCs from patients with RA. (A, C) Chromosomal localization of differentially expressed circRNAs in PBMCs from patients with RA compared with healthy controls (A) and patients with OA (C). (B, D) The average length of differentially expressed circRNAs in PBMCs from patients with RA compared with healthy controls (B) and patients with OA (D).

**Figure 2.**
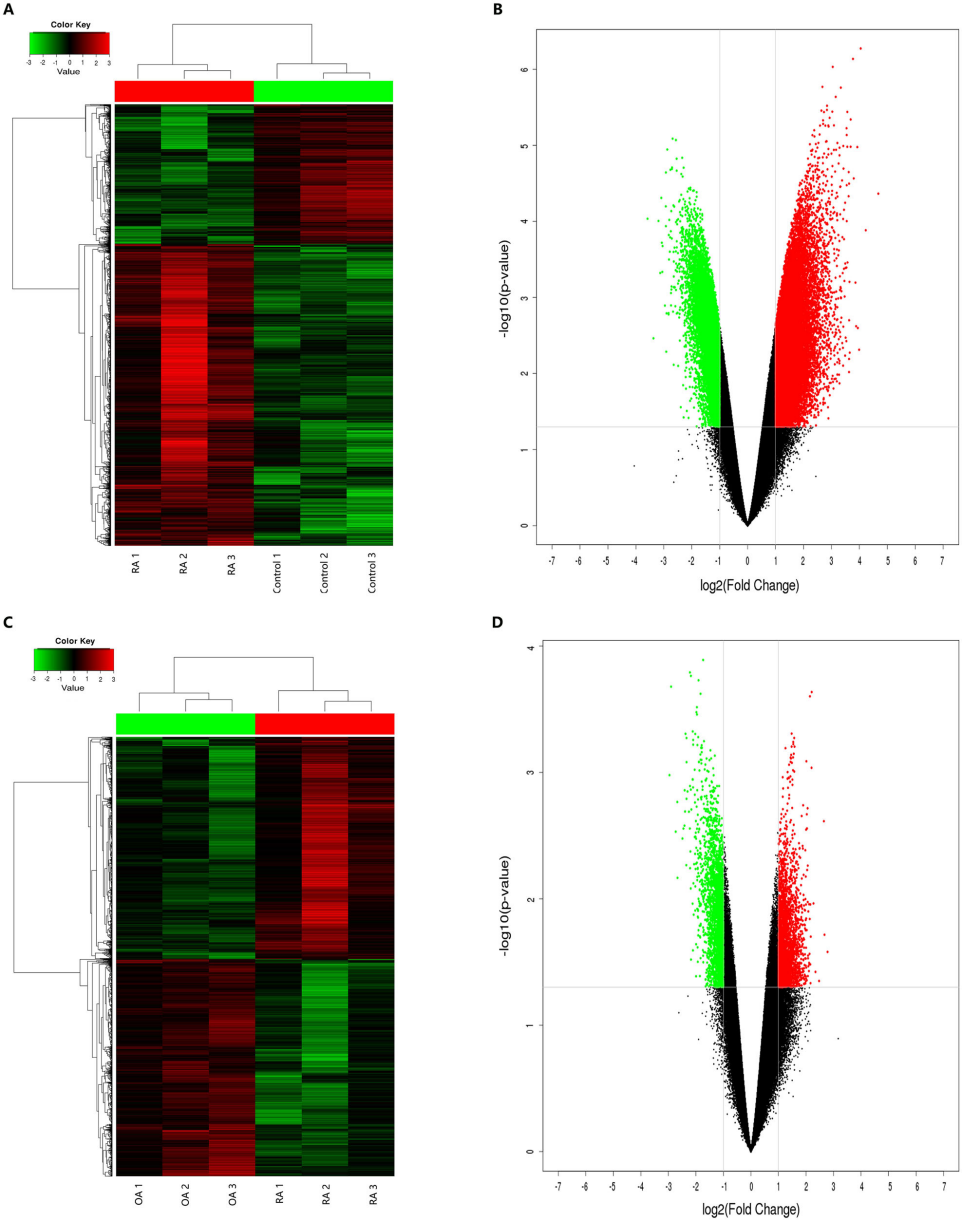
Differentially expressed circRNAs in PBMCs from patients with RA compared to healthy controls and patients with OA. (A, C) Hierarchical cluster analysis of differentially expressed circRNAs in PBMCs from patients with RA compared with healthy controls (A) and patients with OA (C). Rows represent circRNAs while columns represent samples. Red and green indicate expression values of upregulation and downregulation, respectively. (B) and (D): Volcano plots showing the differentially expressed circRNAs. Red and green indicate more than twofold changes in increased and decreased circRNA levels in patients with RA compared with healthy controls (B) and patients with OA (D), respectively (*p* < 0.05).

A total of 170,340 circRNAs were detected in this microarray analysis. Among these circRNAs, 35,342 were differentially expressed in RA patients compared with healthy controls (fold change ≥ 2 and *p* < 0.05), including 20,357 upregulated circRNAs and 14,985 downregulated circRNAs (Figure 1(A)). Additionally, 6146 circRNAs (3109 circRNAs upregulated and 3037 circRNAs downregulated) were identified as being significant in RA compared with OA using the cut-off criteria of ≥2.0-fold changes and *p* < 0.05 (Figure 1(C)). The DEcircRNAs were widely distributed across almost all human chromosomes, including the sex chromosomes (Figure 1(A,C)). The related length distribution of these circRNAs is shown in Figure 1(B,D). Hierarchical clustering showed that circRNA expression profiles were distinctly different between RA and healthy controls (Figure 2(A)) and between RA and OA (Figure 2(C)). Volcano plot analysis was used to visualize the DEcircRNAs (Figure 2(B,D)). The top 10 upregulated and downregulated circRNAs between RA and healthy controls and between RA and OA are displayed in Tables 2 and 3, respectively.

**Table 2.** The top 10 up-regulated and down-regulated circRNAs in PBMCs from RA patients compared with healthy controls.

Probe name	*p* Value	FC	Chromosome	Spliced length	Best transcript	Gene symbol
*Upregulated*						
hsa_circ_0129296_CBC1	0.03097835	25.6138462	chr5	619	ENST00000401507	KIF2A
hsa_circ_0129297_CBC1	0.03097835	18.7533444	chr5	770	ENST00000401507	KIF2A
hsa_circ_0084310_CBC1	0.03097835	16.5247782	chr8	7298	ENST00000523565	PRKDC
hsa_circ_0075542_CBC1	0.04568037	15.8968058	chr6	314	ENST00000264870	F13A1
hsa_circ_0004417_CBC1	0.03804969	15.4054295	chr1	30103	ENST00000366928	LYPLAL1
hsa_circ_0025945_CBC1	0.03097835	15.2016920	chr12	494	ENST00000426898	ANO6
hsa_circ_0096132_CBC1	0.03760922	14.7674313	chr11	105	ENST00000534336	–
hsa_circ_0090099_CBC1	0.03199472	14.5248363	chrX	566	ENST00000489394	SAT1
hsa_circ_0061397_CBC1	0.03097835	13.9097926	chr21	3750	ENST00000286800	BACH1
hsa_circ_0139658_CBC1	0.03097835	13.6671640	chrX	696	ENST00000434600	LAMP2
*Downregulated*						
hsa_circ_0077567_CBC1	0.03097835	11.9905921	chr6	1464	ENST00000426155	SNX3
hsa_circ_0113483_CBC1	0.04099663	10.3576155	chr1	27013	ENST00000337817	STIL
hsa_circ_0133120_CBC1	0.03097835	9.1400433	chr7	299	ENST00000160373	CTTNBP2
hsa_circ_0136535_CBC1	0.0311048	8.7543678	chr8	1710	ENST00000517495	–
hsa_circ_0026804_CBC1	0.03097835	8.5187515	chr12	682	ENST00000552361	RPS26
hsa_circ_0091075_CBC1	0.03097835	8.4081826	chrX	455	ENST00000414209	–
hsa_circ_0092544_CBC1	0.03106526	8.3104465	chr10	1392	ENST00000428666	WDR96
hsa_circ_0088596_CBC1	0.03097835	8.2501194	chr9	81016	ENST00000473837	MAPKAP1
hsa_circ_0052831_CBC1	0.0336258	7.7853451	chr2	2836	ENST00000254351	SDC1
hsa_circ_0001171_CBC1	0.03097835	7.6172276	chr20	145	ENST00000412321	–

**Table 3.** The top 10 up-regulated and down-regulated circRNAs in PBMCs from RA patients compared with OA patients.

Probe name	*p* Value	FC	Chromosome	Spliced length	Best transcript	Gene symbol
*Upregulated*						
hsa_circ_0098712_CBC1	0.02632323	6.8864860	chr12	377	ENST00000518444	LARP4
hsa_circ_0054223_CBC1	0.01925741	6.3721711	chr2	958	ENST00000505747	THUMPD2
hsa_circ_0000579_CBC1	0.002433971	6.2774995	chr14	27943	ENST00000390548	IGHG1
hsa-circRNA9292-4_CBC1	0.04470502	5.5602086	chr10	216	ENST00000369405	ZDHHC6
hsa_circ_0089902_CBC1	0.03785469	5.0789103	chrX	523	ENST00000380550	OFD1
hsa-circRNA2298-2_CBC1	0.01088847	4.8317506	chr14	407	ENST00000335183	CDKN3
hsa_circ_0053881_CBC1	0.000231457	4.6143102	chr2	1412	ENST00000317907	TTC27
hsa-circRNA13773-35_CBC1	0.000920554	4.5989420	chr3	3574	ENST00000350721	ATR
hsa_circ_0070964_CBC1	0.01993587	4.5115839	chr4	259	ENST00000281142	SCLT1
hsa_circ_0024203_CBC1	0.04652761	4.4826224	chr11	5134	ENST00000278616	ATM
*Downregulated*						
hsa_circ_0037720_CBC1	0.001051801	7.7784678	chr16	1362	ENST00000572232	NMRAL1
hsa_circ_0098003_CBC1	0.000209831	7.4740230	chr12	5460	ENST00000393736	RERG
hsa_circ_0049678_CBC1	0.002946642	6.6635871	chr19	1025	ENST00000360228	CACNA1A
hsa_circ_0030682_CBC1	0.00171535	6.3977960	chr13	519	ENST00000595437	FARP1
hsa_circ_0002557_CBC1	0.006829096	6.3660700	chr13	194	ENST00000595437	FARP1
hsa_circ_0125026_CBC1	0.003338279	5.6180599	chr4	116	ENST00000458497	ALPK1
hsa_circ_0034644_CBC1	0.002783396	5.4768980	chr15	994	ENST00000249749	DLL4
hsa_circ_0077567_CBC1	0.002641892	5.3373514	chr6	1464	ENST00000426155	SNX3
hsa_circ_0051704_CBC1	0.001385484	5.3119516	chr19	880	ENST00000509570	SEPW1
hsa_circ_0140790_CBC1	0.002040522	5.2572451	chrY	114	ENST00000445253	–

### Functional analysis of differentially expressed circRNAs

**Figure 3.**
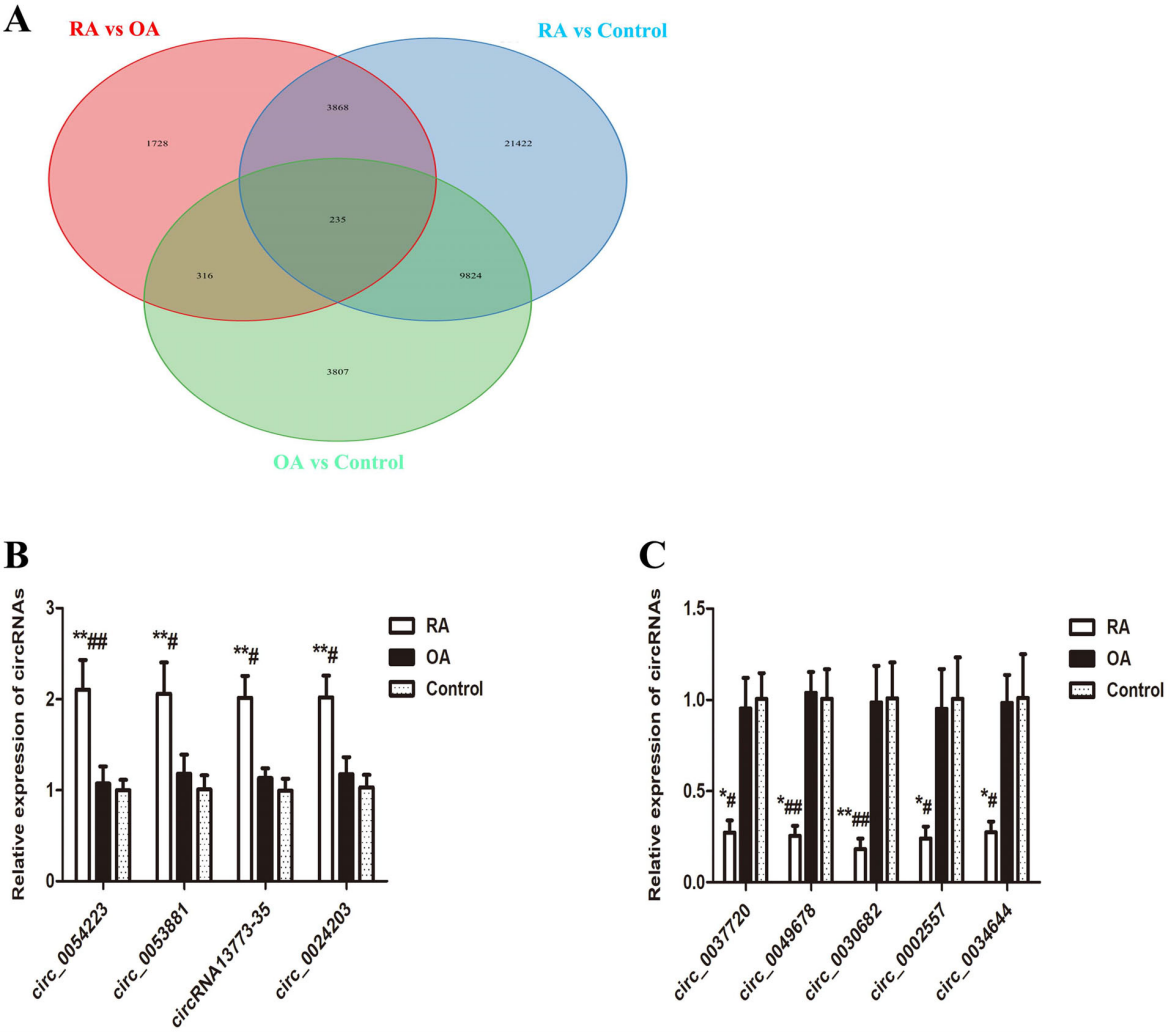
Venn diagram analysis and validation of selected DEcircRNAs by RT-PCR. (A) Venn diagram analysis showing the number of unique and common differentially expressed circRNAs (DEcircRNAs) by comparison between RA, OA and healthy controls. (B, C) Validation of the expression of selected DEcircRNAs by RT-PCR. RT-PCR was performed to analyse the relative expression levels of the four upregulated (B) and the five downregulated (C) circRNAs in PBMCs from 26 RA, 24 OA and 26 healthy controls. The 2^−ΔΔCt^ method was used to calculate the level of these circRNAs relative to the β-actin housekeeping gene. All data are representative of the mean ± SEM. *p < 0.05 compared to the corresponding control group; **p < 0.01 compared to the corresponding control group; ^#^*p* < 0.05 compared to the OA group; ^##^*p* < 0.01 compared to the OA group.

**Figure 4.**
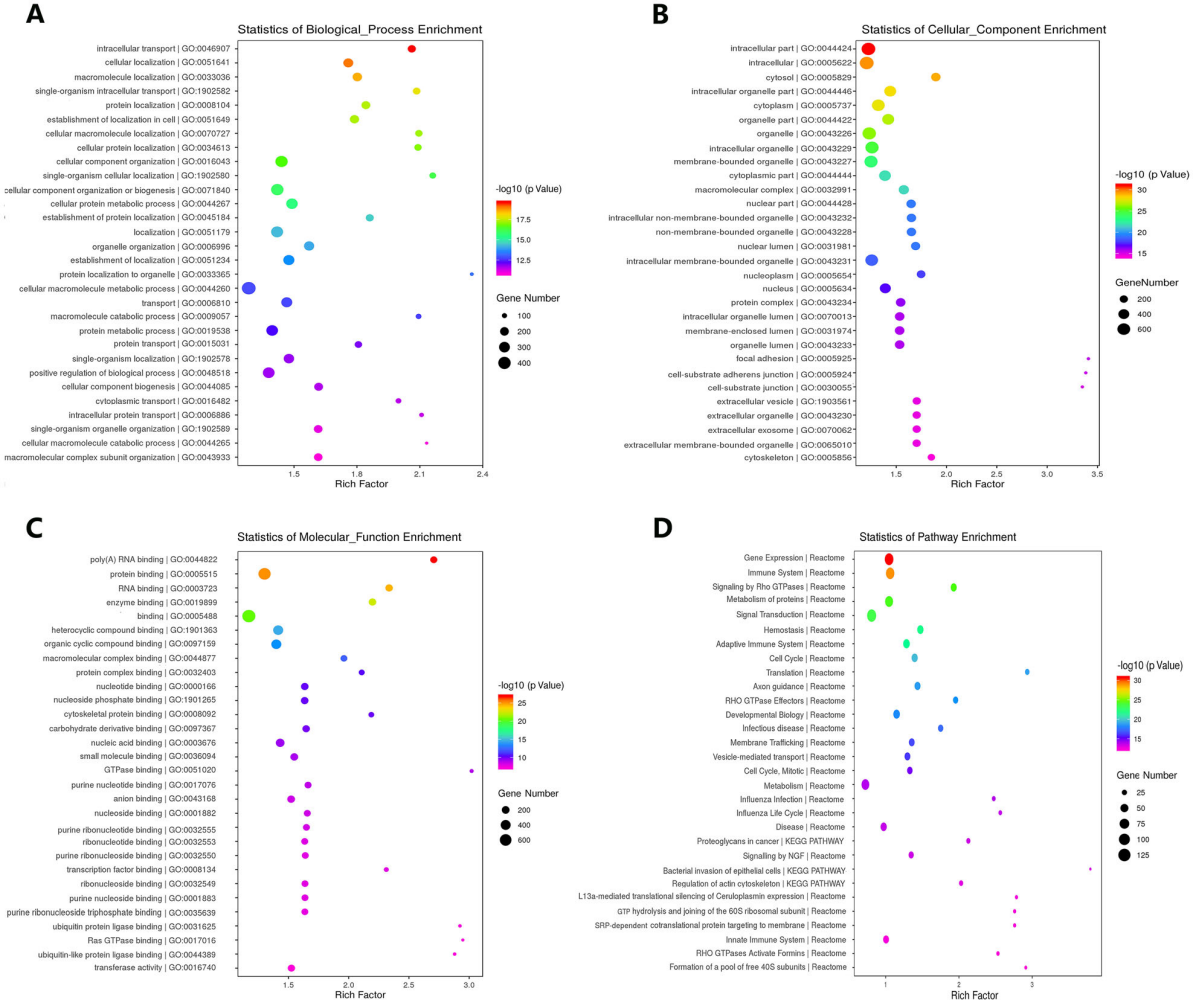
GO annotations and KEGG pathway analysis for host genes of the upregulated circRNAs in PBMCs from patients with RA. (A–C) GO annotation for host genes of these differentially expressed circRNAs (DEcircRNAs) under the theme of biological process (A), cellular component (B) and molecular function (C). (D) KEGG enrichment analysis for host genes of these DEcircRNAs. The *X*-axis shows the enrichment degree of host genes of these DEcircRNAs, and the *Y*-axis shows the category of enriched GO terms and pathways. The area of each node represents the number of enriched host genes. The –log 10 (*p* value) is represented by a colour scale.

**Figure 5.**
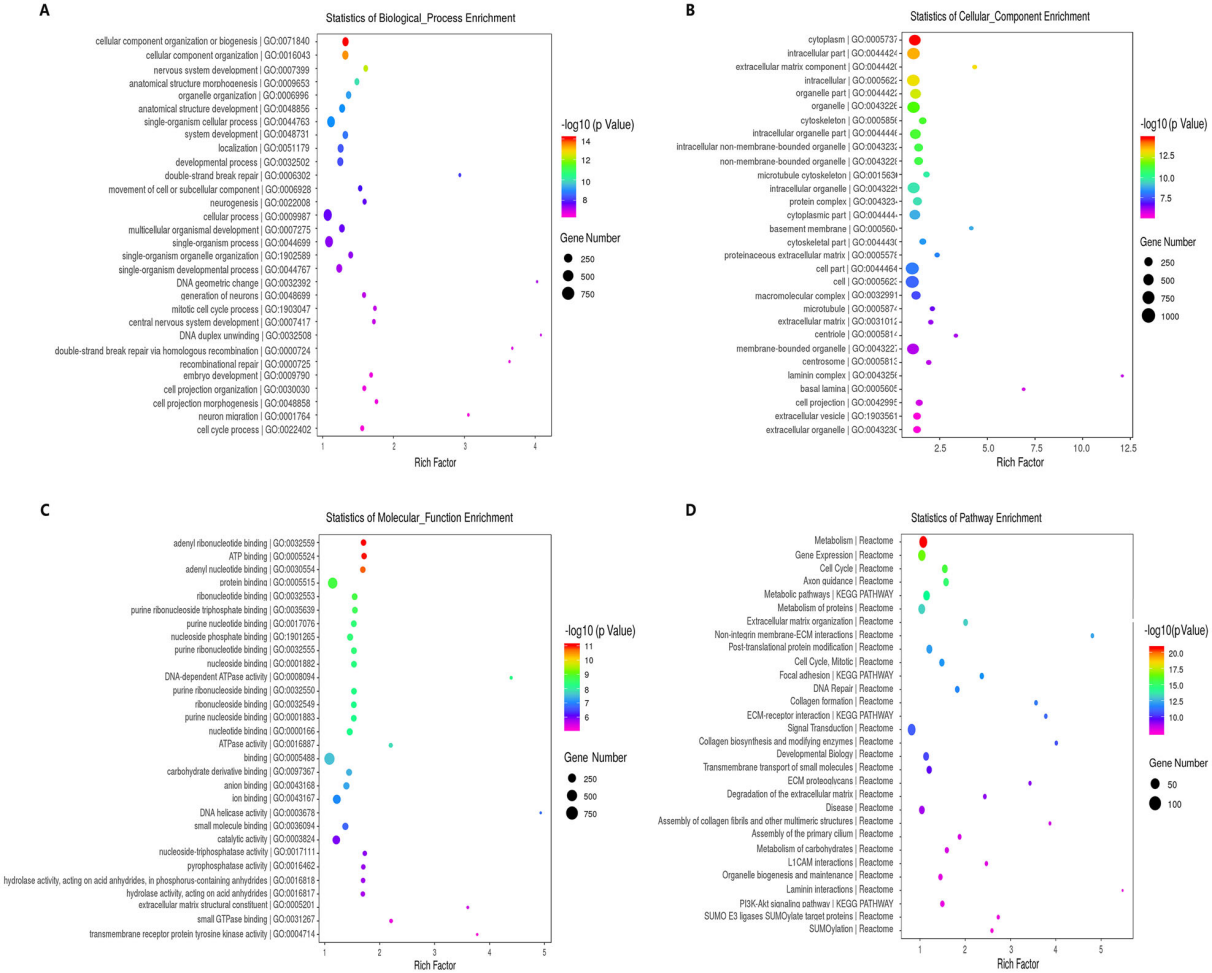
GO annotations and KEGG pathway analysis for host genes of the downregulated circRNAs in PBMCs from patients with RA. (A–C) GO annotation for host genes of these differentially expressed circRNAs (DEcircRNAs) under the theme of biological process (A), cellular component (B) and molecular function (C). (D) KEGG enrichment analysis for host genes of these DEcircRNAs. The *X*-axis shows the enrichment degree of host genes of these DEcircRNAs, and the *Y*-axis shows the category of enriched GO terms and pathways. The area of each node represents the number of enriched host genes. The –log 10 (*p* value) is represented by a colour scale.

We first performed Venn diagram analysis (Figure 3(A)) to find 3868 unique DEcircRNAs that were relevant to the pathological process of RA. Then, GO analysis and KEGG pathway enrichment were used to investigate the putative functions and cellular signalling pathways of these DEcircRNAs in RA. The GO terms are divided into three domains: BP, CC and MF. The top 30 GO processes of each domain and KEGG signalling pathways of unique and upregulated circRNAs in RA are listed in Figure 4, while those of unique and downregulated circRNAs in RA are listed in Figure 5. The results showed that the most significantly enriched KEGG pathways of these upregulated circRNAs in RA were associated with gene expression, the immune system, signalling by Rho GTPases, metabolism of proteins, signal transduction, haemostasis and adaptive immune system. The most significantly enriched KEGG pathways of these downregulated circRNAs in RA were related to metabolism, gene expression, cell cycle, posttranslational protein modification and collagen formation.

### Identification of the circRNA–miRNA–mRNA network

**Figure 6.**
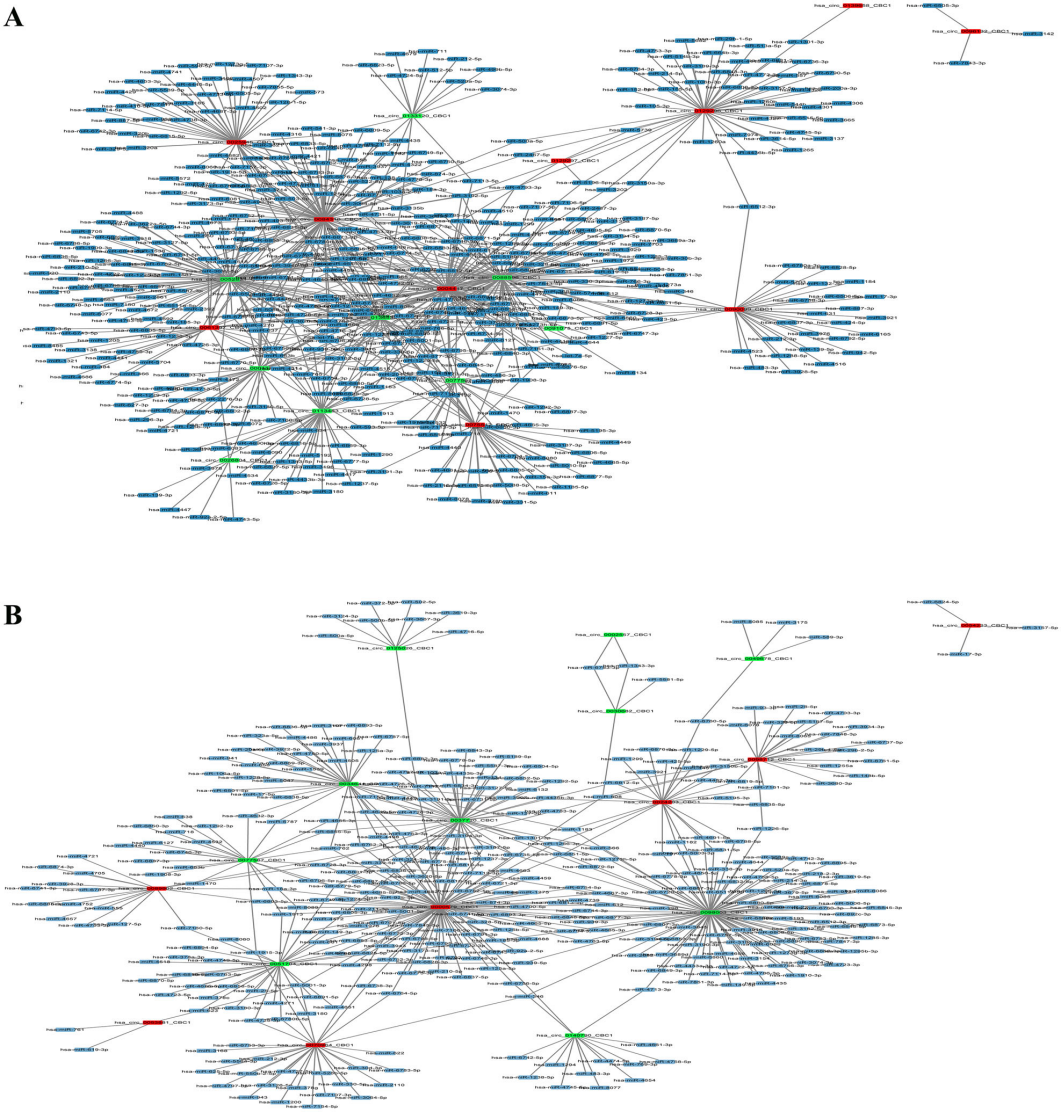
CircRNA-miRNA network analysis in PBMCs of RA patients relative to that of healthy controls and OA patients. TargetScan and MiRanda databases were used to analyse the potential target miRNAs (blue colour) of the top 10 upregulated (red colour) and downregulated (green colour) circRNAs in RA compared with healthy controls (A) and OA (B).

**Figure 7.**
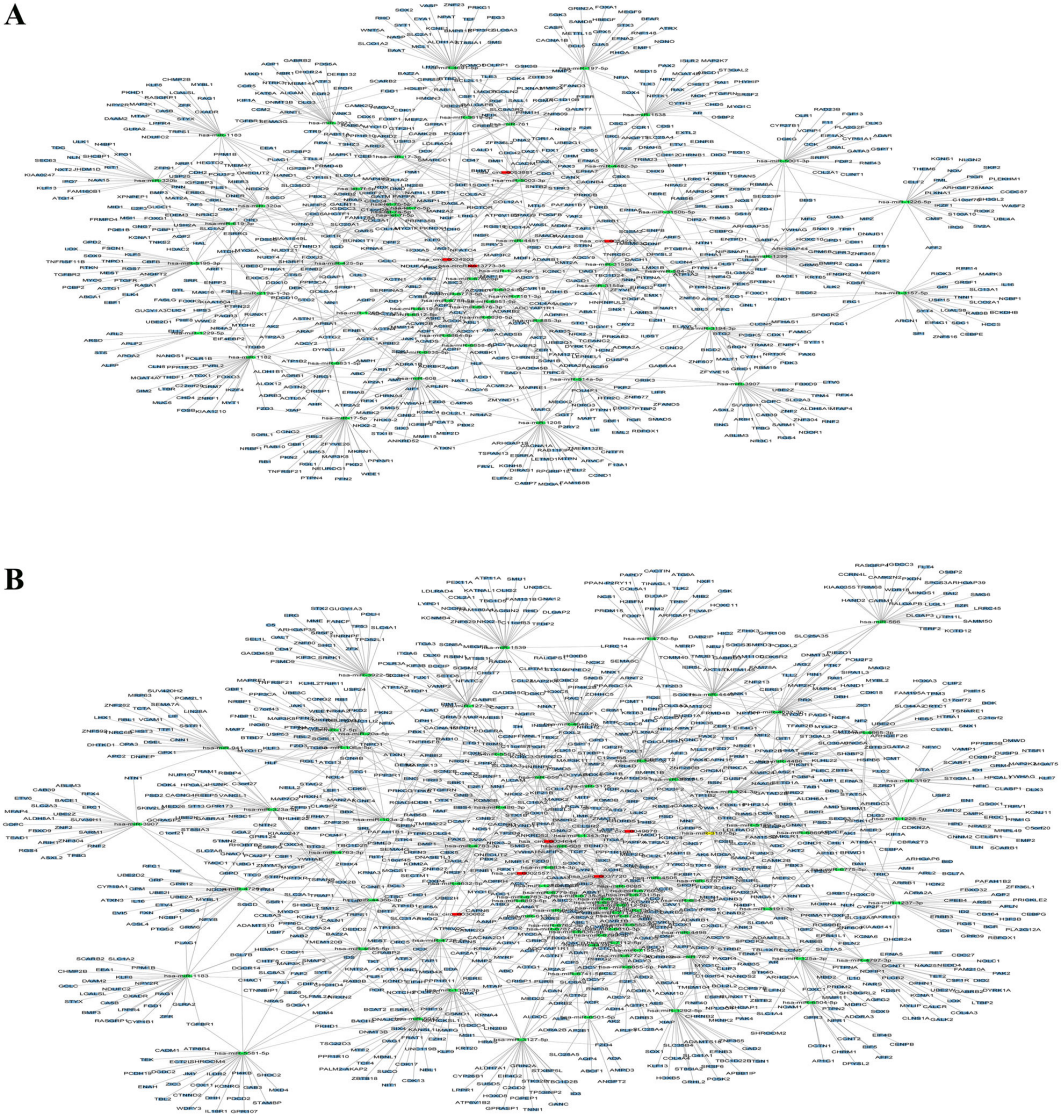
CircRNA–miRNA–mRNA network analysis in PBMCs of RA patients relative to that of OA patients and healthy controls. (A) The ceRNA network was constructed using four upregulated circRNAs (hsa_circ_0054223, hsa_circ_0053881, hsa-circRNA13773-35, hsa_circ_0024203). (B) The ceRNA network was constructed using five downregulated circRNAs (hsa_circ_0037720, hsa_circ_0049678, hsa_circ_0030682, hsa_circ_0002557, hsa_circ_0034644). Blue nodes represent protein-coding mRNAs while miRNAs and circRNAs are shown by green and red nodes, respectively.

**Figure 8.**
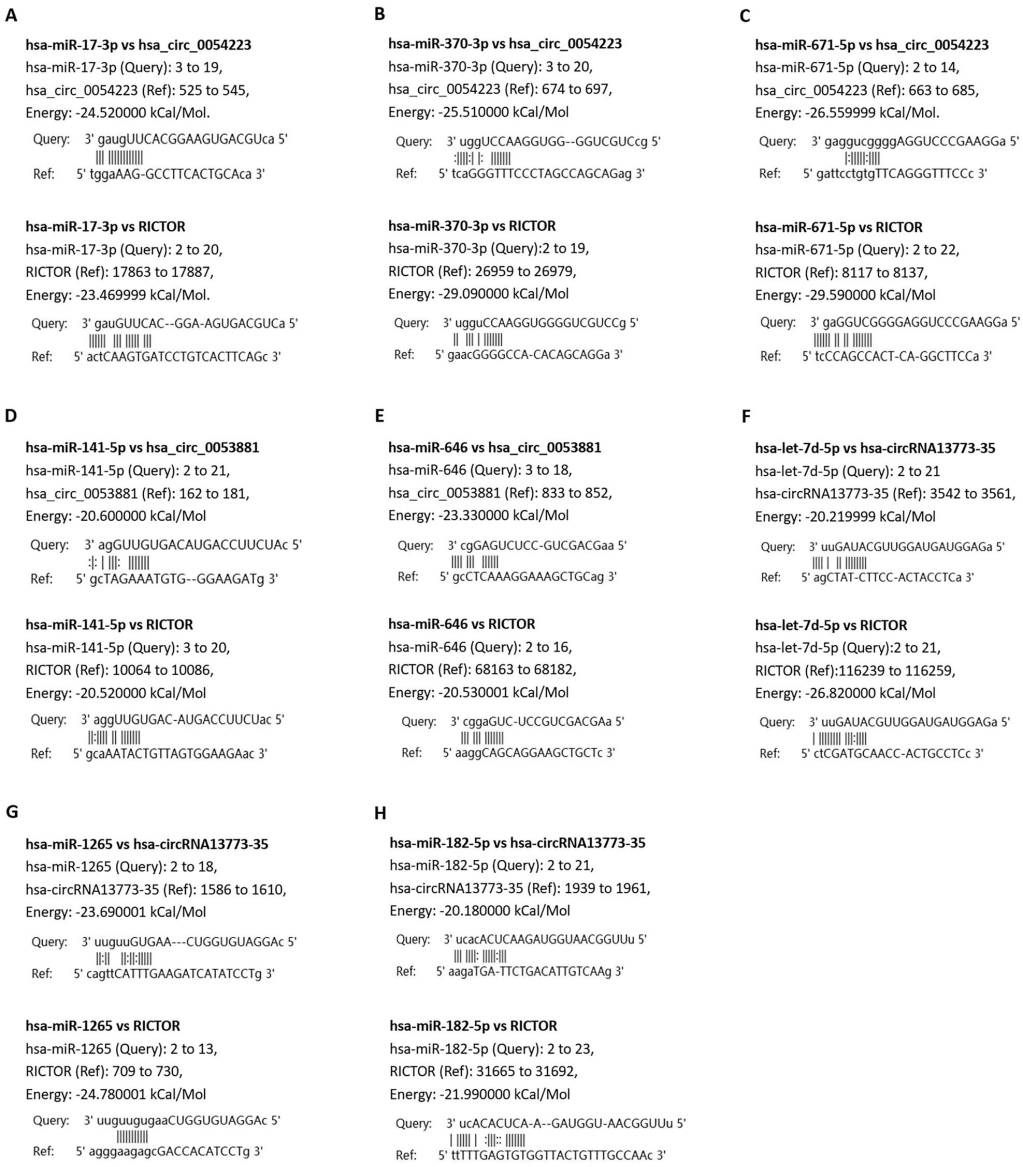
Prediction analysis of complementary bases of circRNA-miRNA-RICTOR interaction. (A–C) Prediction of the binding of hsa_circ_0054223 with hsa-miR-17-3p (A), hsa-miR-370-3p (B) and hsa-miR-671-5p (C) and the binding of complementary bases between RICTOR and the three miRNAs hsa-miR-17-3p (A), hsa-miR-370-3p (B) and hsa-miR-671-5p (C). (D, E) Prediction of the binding of hsa_circ_0053881 with hsa-miR-141-5p (D) and hsa-miR-646 (E) and the binding of complementary bases between RICTOR and the two miRNAs hsa-miR-141-5p (D) and hsa-miR-646 (E). (F–H) Prediction of the binding of hsa-circRNA13773-35 with hsa-let-7d-5p (F), hsa-miR-1265 (G) and hsa-miR-182-5p (H) and the binding of complementary bases between RICTOR and the three miRNAs hsa-let-7d-5p (F), hsa-miR-1265 (G) and hsa-miR-182-5p (H).

CircRNAs have been demonstrated to play a vital role in the transcriptional regulation of genes by functioning as miRNA sponges. Therefore, we identified the miRNAs targeted by the top 10 upregulated (red nodes) and downregulated (green nodes) circRNAs in RA patients compared with healthy controls and OA patients (Figure 6(A,B)). The potential miRNA targets of these DEcircRNAs were analysed by using the TargetScan and MiRanda databases. Then, the network diagram of the association between circRNAs and miRNAs and mRNA was generated by using Cytoscape. As shown in Figure 6(A,B), two of the upregulated circRNAs, hsa_circ_0000579_CBC1 and hsa_circ_0004417_CBC1, could target multiple miRNAs. Furthermore, we performed mutually targeted MRE enrichment (MuTaME) analysis to explore the regulatory relationships among several validated DEcircRNAs, their targeted miRNAs and the relevant mRNAs in PBMCs from RA patients. The circRNA–miRNA–mRNA networks from the upregulated circRNAs (hsa_circ_0054223, hsa_circ_0053881, hsa-circRNA13773-35 and hsa_circ_0024203) and downregulated circRNAs (hsa_circ_0037720, hsa_circ_0049678, hsa_circ_0030682, hsa_circ_0002557 and hsa_circ_0034644) are shown in Figure 7(A,B), respectively. The regulatory function of these DEcircRNAs partially overlapped in this network analysis. Notably, RICTOR was illustrated to be the common targeted mRNA of these upregulated circRNAs. Bioinformatics analysis showed that there were binding sites between the four upregulated circRNAs and the corresponding miRNAs that could also subsequently bind to RICTOR mRNA (Figure 8).

### qRT-PCR validation of microarray data

We performed qRT-PCR to validate the microarray data of 10 randomly selected DEcircRNAs in patients with RA, including five top upregulated circRNAs (hsa_circ_0054223, hsa-circRNA2298-2, hsa_circ_0053881, hsa-circRNA13773-35 and hsa_circ_0024203) and five downregulated circRNAs (hsa_circ_0037720, hsa_circ_0049678, hsa_circ_0030682, hsa_circ_0002557 and hsa_circ_0034644) compared with patients with OA. These 10 circRNAs were also differentially expressed in RA patients compared to healthy controls. We verified nine circRNAs but were unable to detect hsa-circRNA2298-2, suggesting that this circRNA was expressed at a low level or not expressed at all (Figure 3).

### Correlation between the DEcircRNAs and laboratory data of RA patients

Table 4 presents the correlation between DEcircRNAs and various laboratory variables in RA patients. The results showed that the levels of circ_0054223, circ_0053881 and circ_0024203 were positively correlated with ESR (*r* = 0.505, *p* < 0.001; *r* = 0.391, *p* < 0.01; *r* = 0.401, *p* < 0.01, respectively). On the other hand, the levels of circ_0037720, circ_0049678 and circ_0030682 were negatively correlated with ESR (*r*= −0.551, *p* < 0.001; *r*=–0.403, *p* < 0.01; *r*= −0.652, *p* < 0.001, respectively). We also found that the levels of circ_0054223, circ_0053881 and circ_0024203 were positively correlated with CRP (*r* = 0.427, *p* < 0.01; *r* = 0.484, *p* < 0.001; *r* = 0.493, *p* < 0.001, respectively), while the levels of circ_0037720, circ_0049678, circ_0030682 and circ_0002557 were negatively correlated with CRP (*r*= −0.486, *p* < 0.001; *r*= −0.462, *p* < 0.001; *r*= −0.540, *p* < 0.001; *r*= −0.490, *p* < 0.001, respectively). Moreover, RF was positively correlated with the levels of circ_0054223 and circ_0053881 (*r* = 0.408, *p* < 0.01; *r* = 0.425, *p* < 0.01, respectively) and negatively correlated with the levels of circ_0037720, circ_0049678, circ_0030682 and circ_0002557 (*r*= −0.435, *p* < 0.01; *r*= −0.430, *p* < 0.01; *r*= −0.484, *p* < 0.001; *r*= −0.311, *p* < 0.05, respectively).

**Table 4.** Correlation of differentially expressed circRNAs with clinical characteristics of RA patients.

	Age (years)		Disease duration (years)		ESR (mm/h)		CRP (mg/L)		RF (IU/mL)		Anti-CCP (U/mL)		IGA (g/L)		C3 (g/L)	
	*r*	*p* Value	*r*	*p* Value	*r*	*p* Value	*r*	*p* Value	*r*	*p* Value	*r*	*p* Value	*r*	*p* Value	*r*	*p* Value
circ_0054223	–0.119	0.400	–0.173	0.221	0.505	<0.001	0.427	<0.01	0.408	<0.01	0.198	0.159	–0.166	0.241	–0.123	0.384
circ_0053881	–0.184	0.191	–0.075	0.596	0.391	<0.01	0.484	<0.001	0.425	<0.01	0.191	0.174	0.083	0.560	–0.165	0.242
circRNA13773-35	–0.196	0.164	–0.150	0.288	0.169	0.232	0.042	0.765	0.263	0.060	0.197	0.162	–0.098	0.487	0.181	0.199
circ_0024203	0.226	0.107	–0.041	0.774	0.401	<0.01	0.493	<0.001	0.161	0.255	0.173	0.219	0.065	0.645	0.153	0.278
circ_0037720	–0.171	0.226	0.139	0.326	–0.551	<0.001	–0.486	<0.001	–0.435	<0.01	0.145	0.306	0.091	0.519	0.127	0.370
circ_0049678	0.150	0.289	0.162	0.251	–0.403	<0.01	–0.462	<0.001	–0.430	<0.01	–0.216	0.124	0.205	0.145	0.092	0.519
circ_0030682	0.075	0.596	–0.177	0.208	–0.652	<0.001	–0.540	<0.001	–0.484	<0.001	–0.132	0.352	–0.112	0.431	–0.186	0.186
circ_0002557	0.044	0.755	–0.063	0.655	–0.203	0.149	–0.490	<0.001	–0.311	<0.05	–0.182	0.196	–0.088	0.533	0.194	0.168
circ_0034644	0.192	0.174	0.142	0.316	–0.178	0.207	–0.057	0.686	–0.107	0.451	–0.167	0.238	–0.127	0.371	–0.161	0.254

ESR: erythrocyte sedimentation rate; CRP: C-reactive protein; RF: rheumatoid factor; anti-CCP: anti-cyclic citrullinated peptide; IgA: immunoglobulin A; C3: complement C3.

## Discussion

Accumulating data have demonstrated that circRNAs play a critical role in regulating the immune response and inflammation [16]. RA is a chronically progressive autoimmune disease [17], and the mechanisms underlying the role of circRNAs in the pathogenesis of RA remain to be illustrated. In this study, we profiled DEcircRNAs in PBMCs from patients with RA compared to healthy controls and patients with OA. We then performed bioinformatic analyses of these DEcircRNAs and found that these circRNAs were related to metabolic pathways, ECM–receptor interactions, the PI3K–Akt signalling pathway, protein processing in the endoplasmic reticulum, leukocyte transendothelial migration, ubiquitin-mediated proteolysis and so on. We conducted qRT-PCR to verify the expression of 10 selected circRNAs in PBMCs from patients with RA. Further study of these DEcircRNAs could lead to the discovery of novel biomarkers for the early diagnosis of RA or the development of novel therapeutic strategies to prevent disease progression.

Recent advances have led to better diagnostic criteria, improved serologic testing, novel new drugs and better guidelines to manage patients with RA. However, there is no standard definition of early RA [18]. Early RA known as ‘preclinical RA’ clearly begins years to months before it manifests as polyarthritis. A great proportion of these patients with preclinical RA could develop increasing joint pain and swelling in the ensuing months to years, particularly in those with positive anti-citrullinated protein antibody [19]. Since early diagnosis and treatment could prevent the progression of joint damage in 90% of patients with early RA [20], it is important to identify patients with RA as soon as possible [21]. More recent studies have demonstrated that noncoding RNAs primarily including miRNA, long noncoding RNA (lncRNA) and circRNA play a critical role in inflammation and autoimmune regulation and that they are identified as promising biomarkers for the diagnosis and treatment of RA [22]. In this study, we identified DEcircRNAs in PBMCs from RA patients compared with healthy controls and OA patients. Future studies investigating the role of these DEcircRNAs in distinguishing preclinical RA from confirmed RA would be of great interest to understand the potential application of these DEcircRNAs as novel biomarkers of early diagnosis and therapeutic targets in RA patients.

In this study, functional enrichment analyses were also performed to identify the potential roles of DEcircRNAs in the pathogenesis of RA. The GO analysis of the mRNAs targeted by DEcircRNAs showed that CC organization or biogenesis, cellular protein metabolic processes, extracellular matrix component, ATP binding, adenyl ribonucleotide binding, protein binding and protein transport are implicated in the biological and cellular processes related to the pathogenesis of RA. Recently, it has been well recognized that metabolic stress is closely involved in the pathophysiology of systemic autoimmune diseases [23]. For example, glutamine metabolism was demonstrated to have distinct roles in promoting Th17 but constraining Th1 and CTL effector cell differentiation [24]. Another study found that the metabolic stress sensing protein kinase GCN2 played a critical role in regulating the tolerogenic response to apoptotic cells and limiting autoimmunity [25]. Collectively, these aberrantly expressed circRNAs in PBMCs are implicated in many pathophysiological processes of RA, especially those regarding inflammation and immunity, and may be promising biomarkers and therapeutic targets of RA.

The KEGG pathway analysis indicated that these DEcircRNAs might be responsible for the pathophysiology of RA by regulating metabolic pathways, ECM–receptor interactions, the PI3K–Akt signalling pathway, the AMPK signalling pathway, regulation of the actin cytoskeleton, protein processing in the endoplasmic reticulum, leukocyte transendothelial migration, platelet activation, the cAMP signalling pathway and the Ras signalling pathway. All of these pathways are associated with the regulation of immune responses, and previous studies have revealed that both innate and adaptive immune dysregulation are involved in the pathogenesis of RA [26–28]. The AMPK cascade signalling pathway plays a central role in regulating many important pathophysiological processes, including glucose homeostasis, lipid metabolism, mitochondrial biosynthesis and protein synthesis [29], so it has become an attractive therapeutic target for many diseases. We previously found that the dopamine D3 receptor on mast cells could alleviate inflammation in mouse RA through the mTOR/AKT/AMPK signalling axis [30]. Interleukin (IL)-17 is a key regulator of immune and inflammatory responses [31], and the PI3K/Akt signalling pathway was implicated in the overproduction of IL-17 in RA patients [32]. The activation of the PI3K/Akt signalling pathway was also responsible for the neoangiogenesis of synovial tissues in patients with RA [33]. However, further study is needed to confirm this gene pathway in the pathologic process of RA.

As a new type of noncoding RNA, circRNAs function as competing endogenous RNAs of their targeted miRNAs to upregulate the expression of target genes [34,35]. In this study, we also constructed a ceRNA network to further explore the miRNA sponge role of circRNA in regulating gene expression. From the visible circRNA–miRNA–mRNA network of our results, it can be concluded that circRNAs may regulate mRNA expression by sponging one or several targeted miRNAs. For example, Rictor has been demonstrated to have an important effect on immune responses by regulating the differentiation and function of immune cells such as T cells, B cells and macrophages [11,36,37]. Therefore, Rictor plays a key role in the pathogenesis of autoimmune diseases. In this study, it was shown that hsa_circ_0054223_CBC1, hsa_circ_0053881_CBC1, hsa-circRNA13773-35_CBC1 and hsa_circ_0024203_CBC could all bind to their corresponding miRNAs to affect the expression of RICTOR. The interactive regulation among circRNAs, miRNAs and mRNAs indicated in our study could provide a novel perspective for the pathogenesis of RA.

There were some limitations in this study. First, we had relatively few RA patients for the real-time quantitative PCR (RT-qPCR) verification of the DEcircRNAs, and therefore, it is necessary to further validate our results in a larger RA cohort. We need to corroborate the association of these circRNAs with the disease activity of RA in our future study. We will also investigate the translational potential of these DEcircRNAs in the diagnosis, prognosis and therapeutic evaluation of RA patients in our future study, on account of these findings. Second, the function of the DEcircRNAs was only based on bioinformatics analysis in this study. We should perform more experiments by using methods of controlling the expression of these circRNAs to verify the precise functions of the DEcircRNAs in RA. Finally, PBMCs contain multiple cell types, including lymphocytes, neutrophils, monocytes and NK cells. Consequently, it may be worthwhile to clarify the expression profiles of circRNAs in different types of PBMCs, which deserves to be done in our future study. Given all these points, it might be necessary to investigate novel strategies for the early diagnosis, prognosis and evaluation of therapies by targeting these DEcircRNAs found in this study as laboratory biomarkers of RA and their relevant key signalling pathways in our future study.

## Data Availability

The datasets used and analysed during this study are available from the corresponding author on reasonable request.
